# Circadian misalignment increases mood vulnerability in simulated shift work

**DOI:** 10.1038/s41598-020-75245-9

**Published:** 2020-10-29

**Authors:** Sarah L. Chellappa, Christopher J. Morris, Frank A. J. L. Scheer

**Affiliations:** 1 Medical Chronobiology Program, Division of Sleep and Circadian Disorders, Departments of Medicine and Neurology, Brigham and Women’s Hospital, Boston, MA 02115 USA; 2 Division of Sleep Medicine, Department of Medicine, Harvard Medical School, Boston, MA 02115 USA

**Keywords:** Neuroscience, Circadian rhythms and sleep

## Abstract

Night shift work can associate with an increased risk for depression. As night workers experience a ‘misalignment’ between their circadian system and daily sleep–wake behaviors, with negative health consequences, we investigated whether exposure to circadian misalignment underpins mood vulnerability in simulated shift work. We performed randomized within-subject crossover laboratory studies in non-shift workers and shift workers. Simulated night shifts were used to induce a misalignment between the endogenous circadian pacemaker and sleep/wake cycles (circadian misalignment), while environmental conditions and food intake were controlled. Circadian misalignment adversely impacted emotional state, such that mood and well-being levels were significantly decreased throughout 4 days of continuous exposure to circadian misalignment in non-shift workers, as compared to when they were under circadian alignment (interaction of “circadian alignment condition” vs. “day”, mood: *p* < 0.001; well-being: *p* < 0.001; adjusted *p*-values). Similarly, in shift workers, mood and well-being levels were significantly reduced throughout days of misalignment, as compared to circadian alignment (interaction of “circadian alignment condition” vs. “day”, mood: *p* = 0.002; well-being: *p* = 0.002; adjusted *p*-values). Our findings indicate that circadian misalignment is an important biological component for mood vulnerability, and that individuals who engage in shift work are susceptible to its deleterious mood effects.

## Introduction

**Figure 1:**
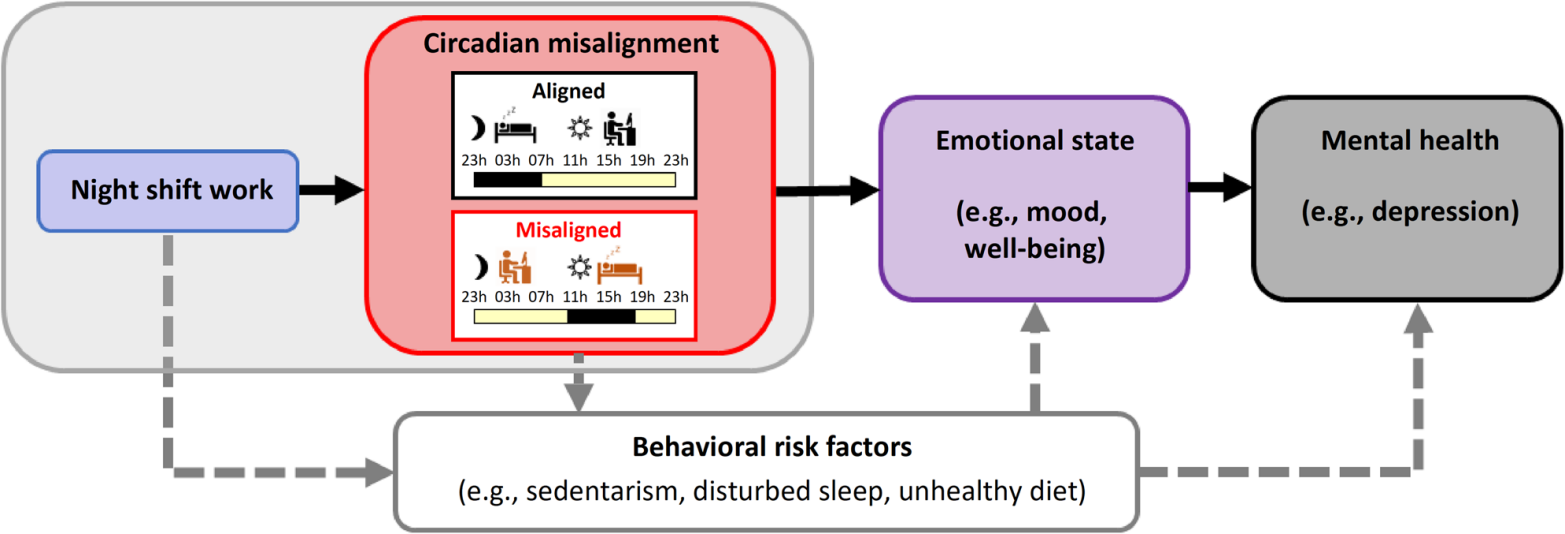
Linking shift work to mood vulnerability. Night shift work may affect emotional state and thereby mental health through circadian misalignment (black solid lines: potential direct effects). Here, circadian misalignment is illustrated by a large ~ 12-h inversion of the sleep/wake cycle and light–dark cycle (red box; aligned: top panel, misaligned: bottom panel), as can occur in night shift work. Shift work also indirectly affects emotional state and mental health through behavioral risk factors (indicated by grey dashed lines).

Depression is a major public health problem and its consequences involve heightened mortality, disability, and morbidity^1^. According to the World Health Organization^2^, the economic toll of depression has an estimated cost to the global economy of ~ US $ 1 trillion/year in lost workforce productivity. Shift work is prevalent throughout the world, with ~ 15% of the workforce performing night shift work in industrialized countries^3^. When so many employees are engaged in shift work, it is important to determine the potential health-related effects of shift work. Most epidemiological data show that shift workers are at a higher risk for depression^4–7^ (but not in^8,9^), such that night shift work is associated with increased psychological strain in the short-term, and in the long-term it may increase the risk of mental illnesses, particularly depression^4,10^. Night shift work can adversely affect mental health though behavioral risk factors, including sedentarism, disturbed sleep, smoking, unhealthy diet, among others^7,11^ (Fig. 1). Night shift workers may differ in their adaptability to shift work, and such variability in the effects of shiftwork maybe due to gender, chronotype, occupation and years of working shifts^12^, which may play a role in their increased risk for mood disruption^13^. Beyond these well-established risk factors, a critical mechanism for the adverse effects of night shift work on emotional state may be *circadian misalignment* (Fig. 1). Shift workers often experience a ‘misalignment’ between their circadian timing system and daily sleep/wake behaviors, with adverse health consequences^14^. As night shift workers typically undergo a large shift in the timing of behavioral and environmental cycles (e.g., sleep/wake, light/dark, fasting/feeding), the resultant misalignment is hypothesized to have a key role in their increased mood vulnerability^15^. Given that shift work is not foreseen to disappear, identifying whether circadian misalignment adversely affects emotional state is needed, as it will help to explain *why* shift workers are at a higher risk for mood disorders.

Circadian factors play a critical role in mood disorders, such that phase-shifts of circadian rhythms relative to the sleep/wake cycle are central to the pathophysiology of depression^16–19^, and conceptual frameworks have linked disruption of circadian rhythms to the genesis of or maintenance of depression in shift workers^20^. While previous studies have reported associations between varying degrees of disrupted circadian phase and the severity of depression^16,21–24^, there is very limited experimental evidence that circadian misalignment per se plays a causal role in disrupting emotional state in either non-shift workers or shift workers. Currently, a single study has identified a potential role of circadian entrainment in mood levels, such that better circadian adaptation to night shift work (i.e., peak melatonin occurred during daytime sleep following 1 week of actual night shift work) associated with better mood levels^25^. Thus, it remains to be established whether circadian misalignment has adverse mood consequences in these individuals. Here, we used randomized, crossover, stringently controlled simulated shift work paradigms to identify whether circadian misalignment affects emotional state, as indexed by subjective levels of mood and well-being, the latter being a construct of mood, stress and physical exhaustion^26.^ Two separate experiments were performed with non-shift workers and shift workers. In the first experiment, we tested the prediction that circadian misalignment increases mood vulnerability and hinders well-being, as compared to normally entrained circadian conditions, in non-shift workers. In the second experiment, we investigated whether the adverse mood effects of circadian misalignment also occur in shift workers. Our main prediction was that similar circadian misalignment effects impinge on mood and well-being levels in individuals who engage in shift work, despite years of exposure to this work schedule. Because night shift workers often experience shorter sleep duration, decreased sleep efficiency and greater variability of sleep–wake times^27^ with ramifications for mood and quality of life, we also tested whether the ability to sleep (e.g., sleep efficiency) was associated with mood and well-being during circadian misalignment. Our two experimental predictions allow establishing whether circadian misalignment is an important biological contributor to mood vulnerability in shift work settings.

## Methods

### Human subjects

The protocol was approved by the institutional review board at the Partners Human Research Committee, performed in accordance with the principles of the Declaration of Helsinki, and participants provided written informed consent. Twenty-three male and female participants between the ages of 18 and 45 years completed the study. These individuals were divided into two groups: 14 non-shift workers (mean age ± SD [range]: 28 ± 9 years [20–49 years]; BMI: 25.4 ± 2.6 kg/m^2^ [21–29.5 kg/m^2^]; six women, eight men)(Experiment 1) and nine shift workers (34 ± 8 years [24–48 years]; BMI: 24.2 ± 3.4 kg/m^2^ [19.3–29.3 kg/m^2^]; six women, three men) (Experiment 2). All participants were free from any acute, chronic or debilitating medical condition. Non-shift workers were matched to shift workers who completed the protocol for (1) sex; (2) age (± 5 years); (3) BMI (± 3 kg/m^2^); and (4) sedentary vs. habitually active lifestyle. In Experiment 1, we included a participant removed from a previous cognitive-related publication on the same experiment^28^. This participant was included after assessing whether or not they were an outlier for any of the outcomes reported here. As the inclusion/exclusion of this single participant did not alter any of the mood-related results presented here, they were included in the current study sample. In Experiment 2, we only enrolled shift workers who had permanent night shifts, rotating shifts, or irregular schedules. All participants in Experiment 2 were currently employed shift workers, a minimum of 12 months of consecutive shift work, at least five night shifts/month (night shift here defined as 6 h of work scheduled between 10 p.m. and 8 a.m.), and no shift duration > 12 h. Because the duration of their consecutive shift work experience was longer than 1 year, we deemed participants as chronically exposed to shift work. Two participants’ dim light melatonin onset (DLMOn) showed a phase difference > 4 h between the circadian alignment and misalignment conditions ^29–31^. Thus, we excluded data from these two participants from all mood and well-being analysis due to the unstable timing of their central circadian clock. The demographics for the remaining seven participants are as follows: age 37 ± 7 years old (30–48 years old); sex, three men and four women; and BMI 24.4 ± 3.1 kg/m^2^ (21.0–29.3 kg/m^2^). Furthermore, the remaining seven participants had night work frequency of 12 ± 4 night shifts/month (6–18 night shifts/month), consecutive shift work experience of 5.3 ± 8.8 years (1.3–25.1 years), and lifetime cumulative shift work experience of 6.3 ± 8.5 years (1.5–25.1 years).

Other aspects of these two studies, which were designed to test separate, independent hypotheses, involving metabolic, cardiovascular, energy balance and cognitive function outcomes, have previously been published^28–37^.

#### Experiment 1

**Figure 2:**
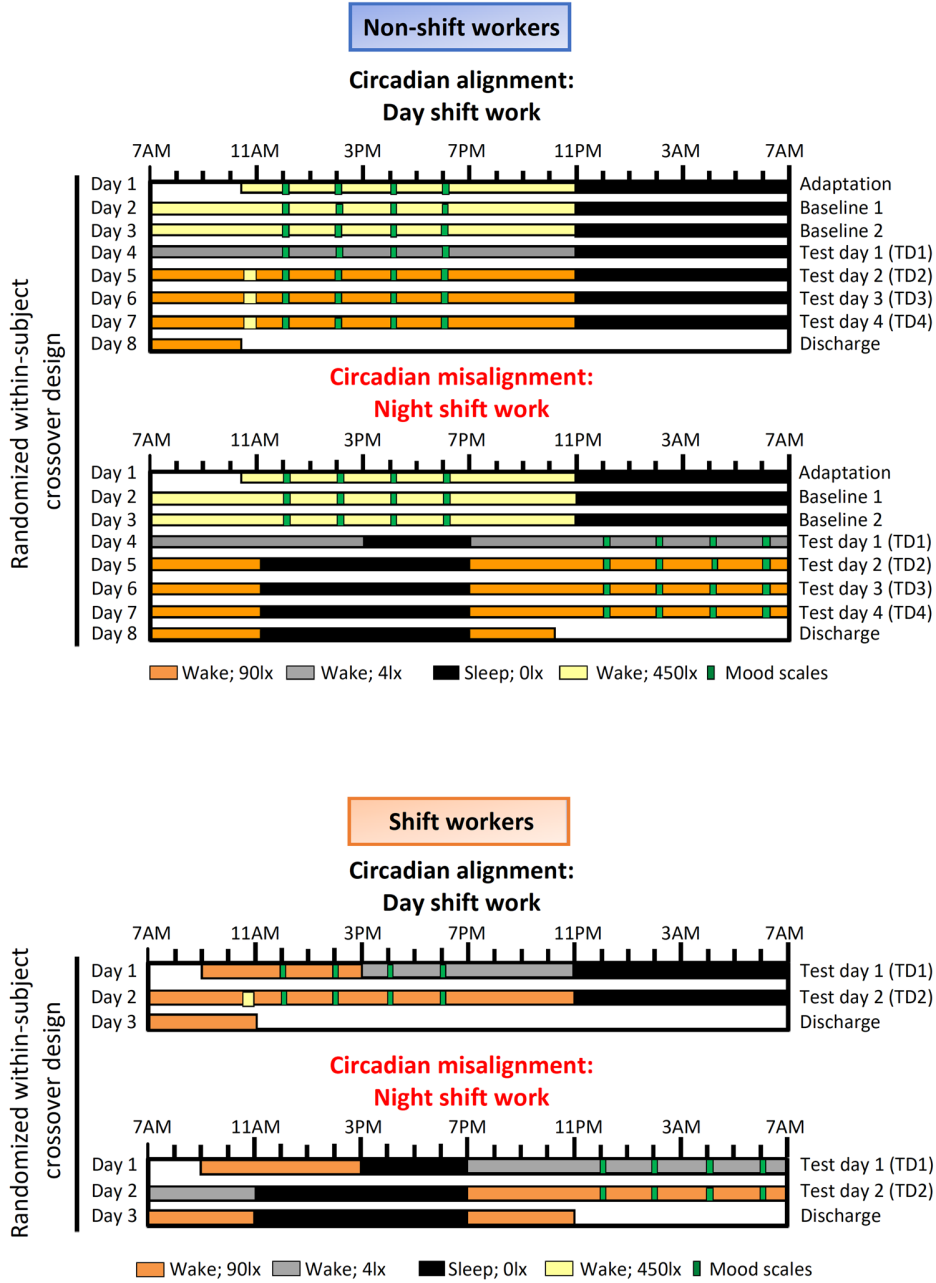
Within-subject, randomized, crossover study design. **Upper panel:** Non-shift workers underwent circadian alignment and misalignment. For alignment condition, scheduled sleep times remained 11 p.m. to 7 a.m. across all days, whereas for the misalignment these timings were inverted by 12 h after Baseline 2 (Day 3). T1–T4 corresponds to test days 1–4. During baseline days for both aligned and misaligned conditions, computerized mood scales were conducted at 12 p.m., 2 p.m., 4 p.m. and 6 p.m. These timings are: (1) outside of the window of sleep inertia effects following awakening; and (2) hours before participant’s scheduled sleep, when they are expected to be alert. While these times were the same throughout the aligned condition, in the misaligned condition the times were inverted by 12 h for T1–T4 relative to clock time, while maintaining the same time relative to the sleep/wake cycle. **Bottom panel:** Shift workers underwent circadian alignment and misalignment conditions that were conceptually similar to Experiment 1, such that scheduled sleep times were maintained between 11 p.m. to 7 a.m. in the alignment condition, whereas the times were inverted by 12 h in the misaligned condition. Both experiments had the same computerized mood scales.

For ~14 days before each laboratory protocol, participants wore an Actiwatch Spectrum (Philips-Respironics) and recorded their bedtimes, wake times, and work schedules in a diary and by reporting the information to a time-stamped voicemail system. Participants were instructed to sleep between 11 p.m. and 7 a.m. on the night before each laboratory protocol to minimize potential sleep debt before the study. Following this ambulatory phase, all participants underwent a randomized, within-participant, crossover design study with two 8-day laboratory protocols. The behavioral and environmental cycles (sleep/wake, fasting/feeding, rest/activity, dark/light) were aligned (circadian alignment) or misaligned (obtained after a rapid 12-h shift of the behavioral cycles) with the endogenous circadian system^37^ (Fig. 2A). The laboratory protocols were separated by 2–8 weeks (mean ± SD, 4 ± 2 weeks). On day 1 in each study protocol, participants were admitted at ∼ 10:30 a.m. and thereafter remained in an individual room throughout each laboratory protocol to ensure stringent environmental condition control. During the circadian alignment condition, each participant’s scheduled sleep and wake times occurred between 11:00 p.m. and 7:00 a.m., respectively, throughout the 8-day laboratory setting. During the circadian misalignment protocol, each participant’s scheduled sleep and wake times occurred between 11:00 p.m. and 7:00 a.m., respectively, for days 1–3. On day 4, their behavioral cycles were shifted by 12-h, and remained as such throughout the entire laboratory protocol. The 12-h shift on day 4 occurred by including an 8-h wake episode and a 4-h sleep opportunity (same sleep opportunity-to-wake ratio [1:2] in both circadian alignment and misalignment conditions). Thereafter, from day 5 to day 8, each participant’s sleep and wake times occurred between 11:00 a.m. and 7:00 p.m. (12-h behavioral cycle inversion), respectively. Light levels were at < 0.02 lx during scheduled sleep as participants remained in individual rooms with no windows, with all lights off, and with all electronic equipment in the room turned off, or—when on—did not emit any light (in case of small LED-light, it was carefully taped off with black electrical tape) during the scheduled sleep episodes. During the scheduled wake episodes of days 1–3, light levels were ∼ 450 lx to enhance circadian entrainment. On day 4, light levels were ∼ 4 lx to allow for assessment of dim-light melatonin onset (DLMO). On days 5–8, light levels were ∼ 90 lx to simulate typical indoor room light intensity under simulated day and night shift work (circadian alignment and misalignment, respectively), with the exception of a brief 30-min 450 lx light exposure to simulate the morning commute. Currently, there is very limited data on light levels in real shift work conditions, e.g.^38^. The 90 lx can result in ~ 50% of the maximum phase shifting capacity and melatonin suppression, and the 450 lx can cause near-maximum phase shifting capacity and melatonin suppression^39^. Moreover, based on^38^, our simulated commute light intensity (450 lx) was close to their reported ~ 550 lx (averaging the two 30-min time windows and for the indoor and outdoor groups). Collectively, our light intensities (in both experiments) are close, but slightly lower than suggested by the limited literature^13^. However, given that thelaboratory  rooms were are white in all directions (including floors), this may have resulted in very similar intensities as compared to the work environment of most shift workers.

#### Experiment 2

For ~14 days before each laboratory protocol, participants wore an Actiwatch Spectrum, recorded their bedtimes, wake times and work schedules in a sleep diary, and reported the information to a time-stamped voicemail system. As for Experiment 1, participants were instructed to sleep between 11 p.m. and 7 a.m. on the night preceding each laboratory protocol to reduce possible sleep debt before commencing the study. Each participant underwent a within-subject, randomized crossover study that comprised two 3-day laboratory protocols^31^ (Fig. 2B). One protocol included a simulated day shift (circadian alignment) and the other a simulated night shift (circadian misalignment). The laboratory protocols were separated by 3–8 weeks (mean ± SD: 5 ± 2 weeks). Minimization was used to reduce imbalance, according to age, sex and BMI. Participants remained in a private laboratory room throughout each protocol to allow for strict control of environmental conditions. Participants were not permitted to exercise while in the laboratory. In the circadian alignment condition, participants’ sleep opportunity occurred from 11 p.m. to 7 a.m. for days 1–3. In the circadian misalignment condition, on day 1 the participants’ sleep/wake cycle was inverted by 12 h by including an 8-h wake episode (beginning at the scheduled time of awakening of 7AM on day 1) plus a 4-h sleep opportunity between 3 and 7 p.m. This 8 h of wake and 4 h of sleep allowed maintaining a 2:1 ratio between scheduled wakefulness and sleep opportunity (same sleep opportunity-to-wake ratio [1:2] in both circadian alignment and misalignment conditions). Participants then stayed awake for 16 h until their next 8-h sleep opportunity that occurred from 11 a.m. to 7 p.m. (maintained until the end of the protocol). Light levels were 90 lx to simulate typical room light intensity, 450 lx for 30-min periods to simulate the morning commute preceding the simulated day shift and following the simulated night shift, 4 lx to permit DLMO assessment, and < 0.02 lx during scheduled sleep. As for Experiment 1, the DLMO assessment allowed identifying whether participants had stable timing of their central circadian clock, whereby their DLMO would not drastically differ between the circadian alignment and misalignment conditions  (< 4 h as per^31^). Experiment 2 was shorter in duration as compared to Experiment 1 to increase feasibility and to minimize potential dropouts of participants with shift work.

### Outcome measures

In both experiments, mood was assessed during computerized test sessions, which were distributed in a time window occurring 5–11 h after scheduled wakefulness on all days, thus avoiding potential subjective impairment due to sleep inertia (typically up to 4-h after awakening)^40^. Key affective components (e.g., mood and well-being) were assessed during these computerized test sessions using a 100-mm bipolar visual analogue general health/well-being scale (VAS; from 0 to 100) to instantaneously detect participants perceived mood and well-being levels^41^. For the assessment of mood, we used the VAS question “happy/sad” (where sad = 0 and happy = 100). Participants had the following instruction presented on the computer screen “move the pointer to the place on the line that is most appropriate to the intensity of the stated symptom as you are experiencing it NOW”. Participants were requested to place a vertical mark on the VAS ranging from 0 to 100 mm. Well-being was defined as a composite score averaged over three items: “happy/sad”, “calm/stressed” (where calm = 0 and stressed = 100; scale was inverted for data analyses), and “energetic/physically exhausted” (where energetic = 0 and physically exhausted = 100; scale was inverted for data analyses). These VAS-derived emotional state measurements have been validated in previous laboratory studies^25,26,42^, and can be reliably used for sequential serial time measurements due to the absence of ceiling effects^26,42^. We did not use the PANAS or the POMS, which allow for the multidimensional assessment of mood (e.g., arousal and valence), because these affective scales cannot be used for sequential serial time measurements (single measurements are most typical when using these scales, for which they were originally designed). Because mood and well-being have been shown to fluctuate with time-of-day^25,26,42^, we therefore opted for using the VAS which is well-validated for such serial time measurements. Sleep was recorded using polysomnography (Vitaport; TEMEC Instruments, The Netherlands)—in accordance with the American Academy of Sleep Medicine recommendations^43^. In Experiment 1, sleep was recorded during sleep periods 1, 4, and 6 in the circadian alignment conditionand during sleep periods 1, 5, and 7 in the circadian misalignment condition. In Experiment 2, sleep was recorded during sleep opportunity 1 in the circadian alignment condition and during sleep opportunity 2 in the circadian misalignment condition. Sleep stages were scored visually per 30-s epochs, according to^43^, by a single experienced polysomnography technician, blind to the circadian alignment and misalignment conditions.

### Statistical analysis

Statistical analyses were performed with SAS version 9.4 (SAS Institute, Cary, NC, USA). Statistical tests were conducted for each of the two experiments (non-shift workers vs. shift workers experiments) separately as they addressed different hypotheses. Because of this, the inherent study design differences between the non-shift workers and shift workers experiments (e.g., length of duration of each experiment) and a low number of shift workers (no participants in Experiment 1 were shift workers), we did not a create a single analytic model with shift work as a between-subjects fixed factor or covariate of interest. Analyses were carried out using mixed-model analyses of variance for repeated measures (PROC MIXED, SAS) with main factors “circadian alignment condition” (aligned vs. misaligned), “time since wake” (5 h, 7 h, 9 h and 11 h), “day”, the two-way interaction of “circadian alignment condition” vs. “day”, and the two-way interaction of “circadian alignment condition” vs. “time since wake”. Participant was included as a random factor, and contrasts were assessed with the LSMEANS statement. Missing data were not included in the analyses. To control overall type I error in null hypothesis testing when conducting multiple comparisons, *p*-values were adjusted using False Discovery Rate (FDR)^44^ on all mood measures. Sleep was treated as a covariate of interest in these analyses, as disrupted sleep has been implicated with worsened mood^27^. Lastly, we applied linear regression models to test whether the ability to sleep (sleep efficiency) is associated with mood and well-being under both circadian conditions. For the analyses in Experiment 2, because of the limited sample size, we did not include years of consecutive shift work nor the work hours per week in our models for emotional state as potential covariates of interest. Unless specified, data are presented as mean ± SEM. Significance for all statistical tests was set as *p* < 0.05.

## Results

**Figure 3:**
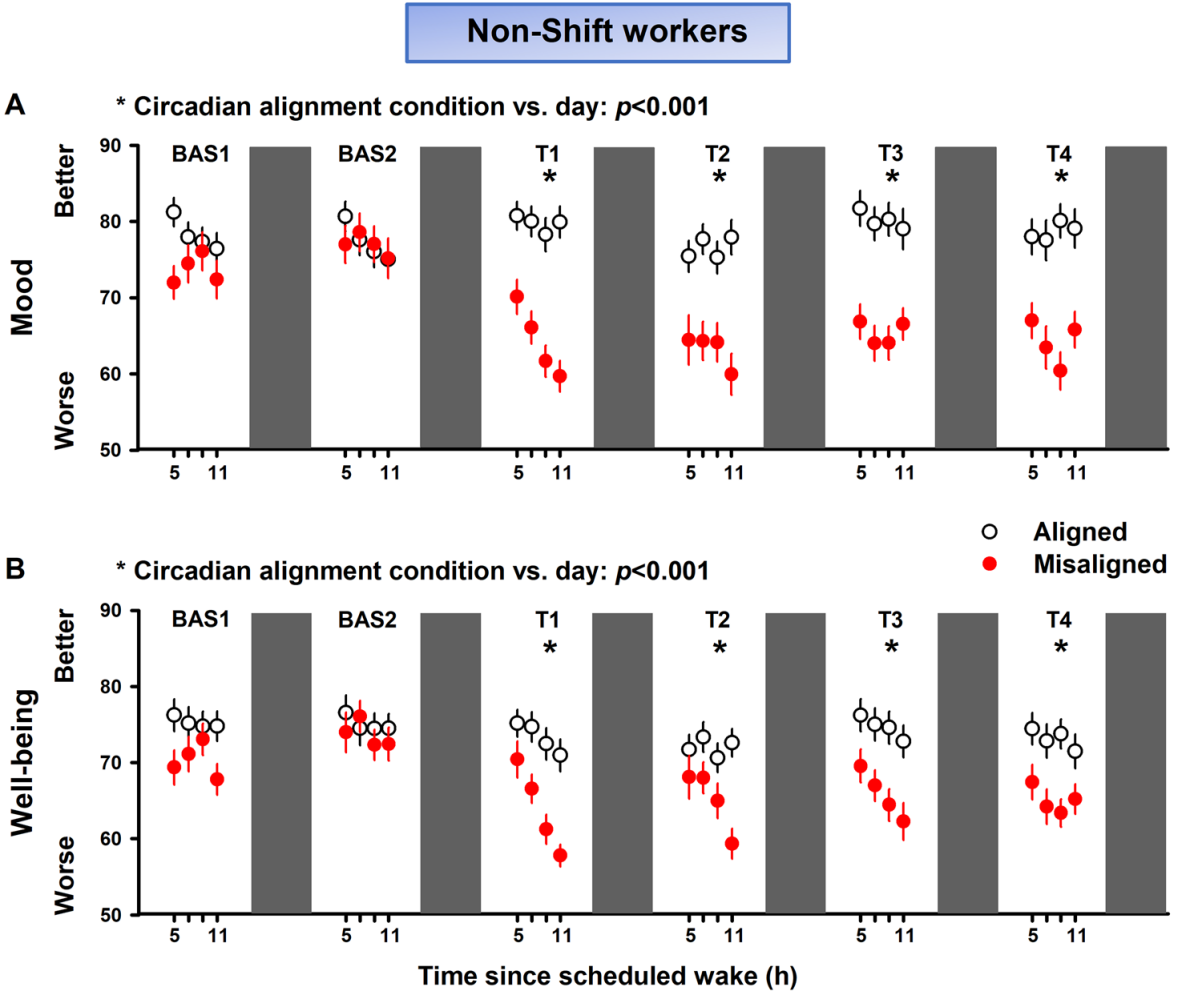
Circadian misalignment affects emotional state in non-shift workers. Circadian misalignment (red circles) influences emotional state, as evidenced by the significantly lower (**A**) mood and (**B**) well-being profiles in non-shift workers (n = 14), as compared to when the same individuals were exposed to days of circadian alignment (black circles). Data correspond to mean and standard error of mean; *p < 0.05 (interaction of “circadian alignment condition” vs. “day”, following FDR adjustment; see “Results” for details).

In the first experiment, we tested the prediction that circadian misalignment increases mood vulnerability and hinders well-being, as compared to circadian alignment, in non-shift workers. Our mixed-model analyses indicated no significant effects for “time since wake” (F_3,641_ = 2.25, *p* = 0.09; FDR adjusted *p*-values) nor the interaction of “circadian alignment condition” vs. “time since wake” (F_3,641_ = 2.18, *p* = 0.11; FDR adjusted *p*-values). Mood significantly varied by  “circadian alignment condition” (F_1,641_ = 223.01, *p* < 0.001; FDR adjusted *p*-values), “day” (F_5,641_ = 10.53, *p* < 0.001; FDR adjusted *p*-values), and by the interaction of “circadian alignment condition” vs. “day” (F_5,641_ = 13.88, *p* < 0.001; FDR adjusted *p*-values). The interaction effect indicated that circadian misalignment significantly decreased mood levels throughout 4 days of continuous exposure to simulated night work in non-shift workers, as compared to when the same individuals were under circadian alignment (Fig. 3A). Well-being significantly varied by “circadian alignment condition” (F_1,636_ = 148.94, *p* < 0.001; FDR adjusted *p*-values), “day” (F_5,636_ = 12.26, *p* < 0.001; FDR adjusted *p*-values), “time since wake” (F_3,636_ = 9.64, *p* < 0.001; FDR adjusted *p*-values). The interaction effect of “circadian alignment condition” vs. “time since wake” did not yield significances (F_5,636_ = 1.97, *p* = 0.27**;** FDR adjusted *p*-values), whereas we observed a significant interaction effect of “circadian alignment condition” vs. “day” (F_5,636_ = 5.47, *p* < 0.001**;** FDR adjusted *p*-values). This interaction effect indicated that circadian misalignment significantly decreased well-being levels throughout 4 days of continuous exposure to simulated night work in non-shift workers, as compared to when they were under circadian alignment (Fig. 3B).

**Figure 4:**
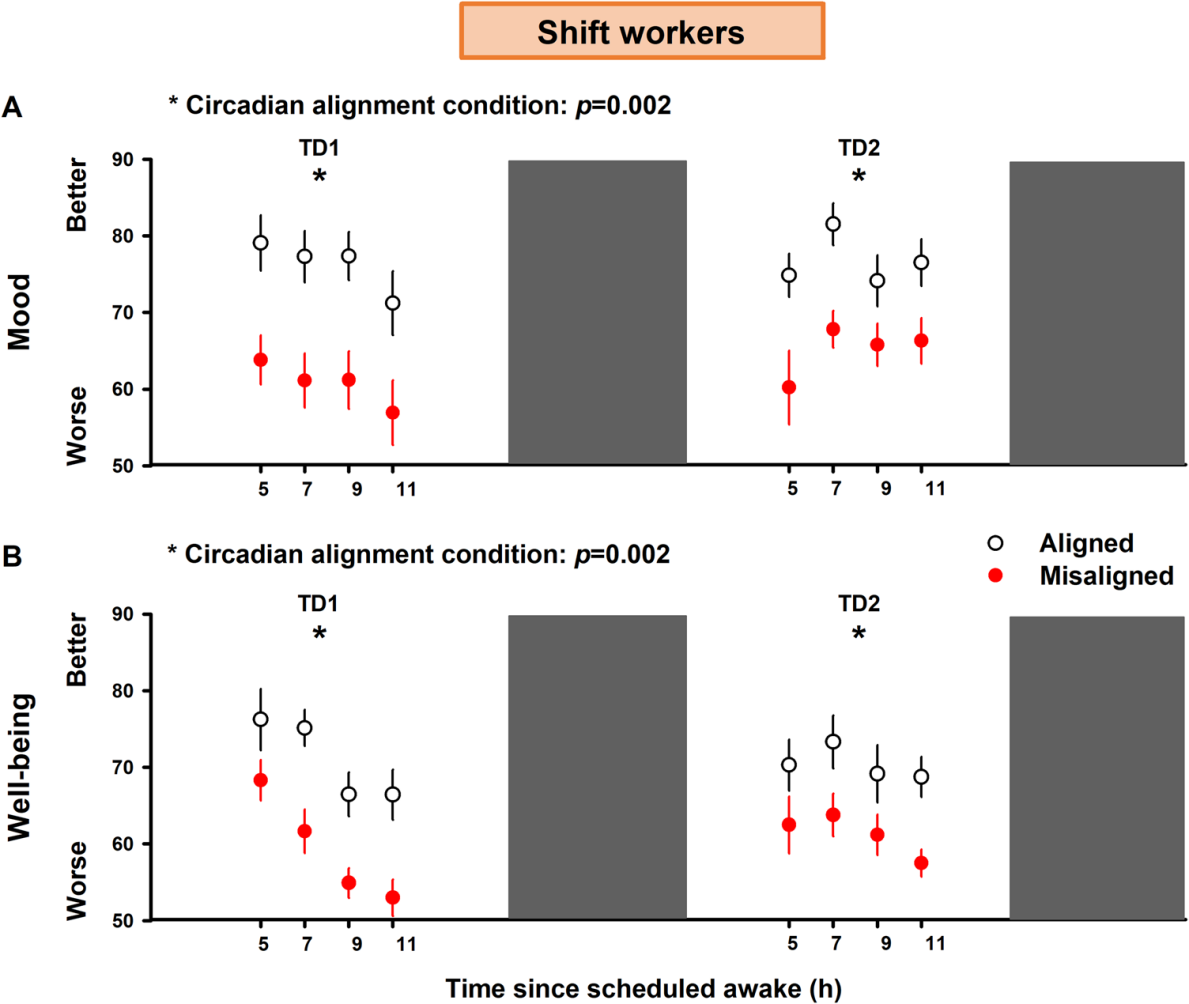
Circadian misalignment affects emotional state in shift workers. Circadian misalignment (red circles) negatively affects emotional state, as evidenced by the significantly lower (**A**) mood and (**B**) well-being profiles in shift workers (n = 7), as compared to when the same individuals were exposed to days of circadian alignment (black circles). Data correspond to mean and standard error of the mean; *p < 0.05 (effect of “circadian alignment condition”, following FDR adjustment; see “Results” for details).

In the second experiment, we tested the prediction that circadian misalignment adversely affects mood and well-being in shift workers. Our mixed-model analyses indicated no significant effects for “time since wake” (F_3,91_ = 0.78, *p* = 0.57; FDR adjusted *p*-values) nor the interaction of “circadian alignment condition” vs. “time since wake” (F_3,91_ = 0.81, *p* = 0.71; FDR adjusted *p*-values). Mood significantly varied by “circadian alignment condition” (F_1,91_ = 85.45, *p* = 0.002; FDR adjusted *p*-values), “day” (F_1,91_ = 34.42, *p* = 0.002; FDR adjusted *p*-values), and by the interaction of “circadian alignment condition” vs. “day” (F_1,91_ = 49.31, *p* = 0.002; FDR adjusted *p*-values). In a similar vein as for the non-shift workers, circadian misalignment significantly decreased mood levels in shift workers, as compared to when they were under circadian alignment (Fig. 4A). Furthermore, well-being significantly varied by “circadian alignment condition” (F_1,91_ = 54.66, *p* = 0.002; FDR adjusted *p*-values), “day” (F_1,90_ = 7.31, *p* = 0.01; FDR adjusted *p*-values), and “time since wake” (F_3,90_ = 3.27, *p* = 0.02; FDR adjusted *p*-values). The interaction effect of “circadian alignment condition” vs. “time since wake” did not yield significances (F_1,90_ = 1.95, *p* = 0.21**;** FDR adjusted *p*-values), while we observed a significant interaction effect of “circadian alignment condition” vs. “day” (F_1,90_ = 13.95, *p* = 0.002**;** FDR adjusted *p*-values). Accordingly, circadian misalignment significantly decreased well-being levels in shift workers, as compared to when they were under circadian alignment (Fig. 4B).

**Figure 5:**
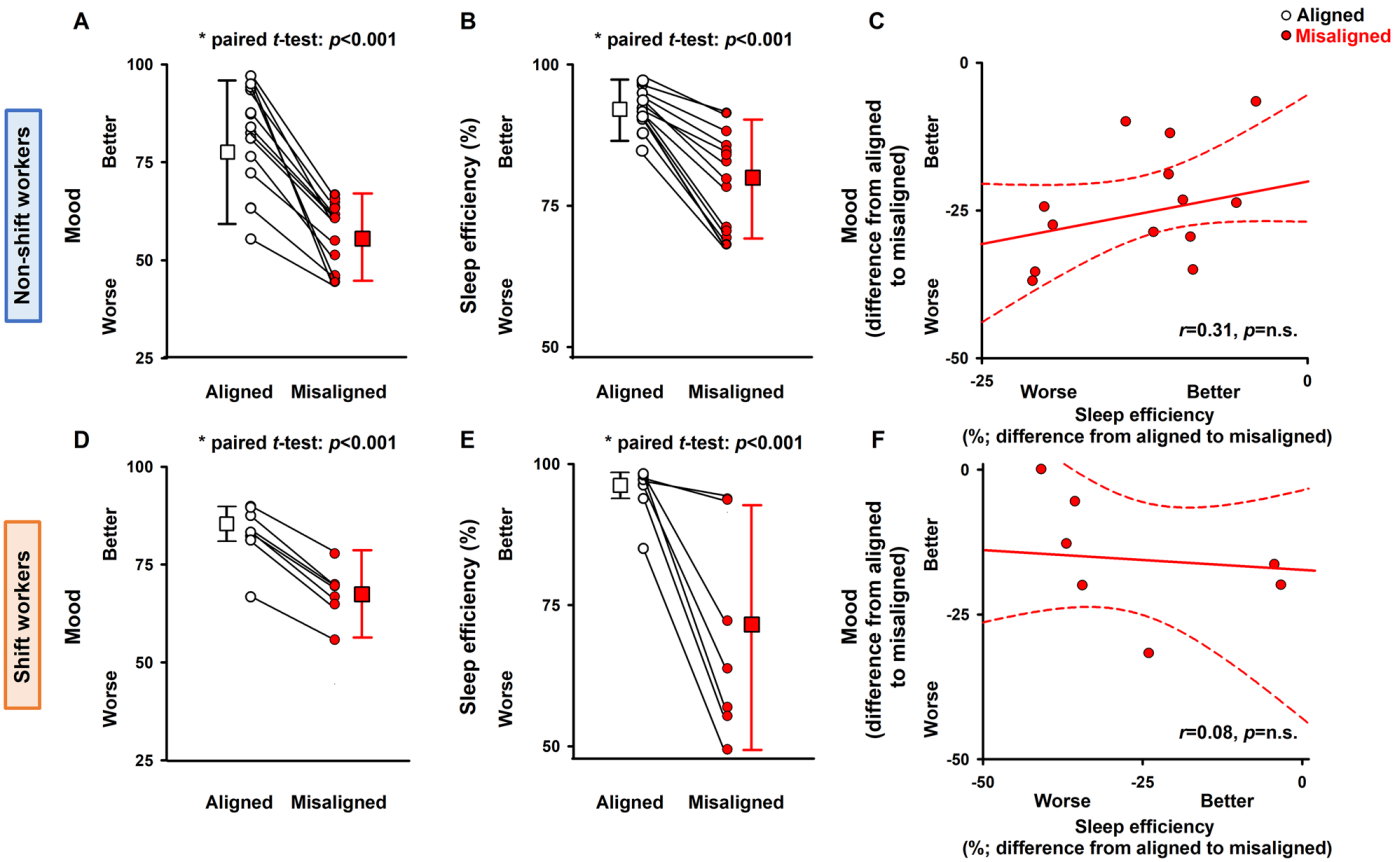
Sleep efficiency does not predict mood and well-being impairment during circadian misalignment. (**A**–**C**) In non-shift workers (n = 14), mood levels (**A**) and sleep efficiency (**B**) were significantly reduced during circadian misalignment as compared to circadian alignment. (**C**) Linear regression models indicate that sleep efficiency did not predict mood levels in the circadian alignment or misalignment conditions. (**D**–**F**) In shift workers (n = 7), mood levels (**D**) and sleep efficiency (**E**) were significantly reduced during circadian misalignment. (**F**) Linear regression models indicate that sleep efficiency did not predict mood levels in either circadian conditions (difference from circadian alignment to misalignment). Data presented as circles indicate individuals, and connecting lines correspond to results from the same individual for circadian alignment and misalignment. Squares and whiskers correspond, respectively, to median and interquartile ranges (minimum and maximum quartiles [1.5 × interquartile range]) to illustrate data dispersion; *p*-values: significance of the paired *t*-tests for **A**,**B**,**D** and **E** and for the linear regression analyses shown in **C** and **F**.

Lastly, we tested whether the ability to sleep (sleep efficiency) was associated with mood and well-being in both experiments. While sleep efficiency significantly decreased with misalignment in both non-shift workers and shift workers, in neither case it was significantly associated with mood (Fig. 5; linear regression models) nor with well-being (data not shown). We also performed linear regression of other sleep markers (sleep duration, slow-wave sleep, REM sleep and wake duration after sleep onset) with mood and well-being, and did not observe any significant associations between these variables for either experiment (data not shown).

## Discussion

Our results indicate that circadian misalignment underpins mood vulnerability in shift work settings. Accumulating data suggest that the mechanisms underlying the association between shift work and worsened mood may include disruption of circadian rhythms and (at least in part consequential) sleep alterations^4,7,25^. The endogenous circadian system determines the appropriate timing for sleep and wakefulness, body temperature, heart rate, blood pressure, and hormone levels, such as cortisol and melatonin^45^. Importantly, previous stringently controlled circadian studies have shown that mood and brain activity that potentially underlies mood regulation are under circadian control both in healthy individuals^26,46^ and in patients with  major depressive disorder^47–49^, which suggests that the circadian system is an important biological component of mood regulation^16–19^. Night shift work is at odds with our endogenous circadian system, which is biologically tuned to ensure optimal alertness and mood levels during the biological day and promote sleep during the biological night, even in individuals who engage in shift work for many years^50^. Therefore, shift workers often experience a misalignment between the endogenous circadian timing system and daily behavior/environmental cycles^14^. Chronobiological adaptation of the sleep–wake cycle in those who work night shifts is very frequently inadequate, and such misalignment between the circadian timing system and daily behaviors is a very likely explanation for the adverse health consequences associated with shift work. Prior Forced Desynchrony studies have found both circadian and time-since-wake components to mood, with a nadir in mood levels that corresponds to the nadir in the circadian core body temperature (during the biological night)^51,52^. Mood assessments during our simulated night shift work occurred during the biological night^37^ (in that study, which assessed glucose tolerance in the same two experiments, circadian phase was estimated by cortisol). Since the mood assessments very likely occurred close to the nadir of core body temperature, participants may have been exposed to the lowest levels of the circadian mood rhythm. Furthermore, in patients with delayed sleep phase disorder (DSPD), circadian misalignment may be associated with increased depressive symptoms and DSPD symptom severity^24^, consistent with an interrelationship between circadian misalignment and mood vulnerability. Importantly, our stringently controlled within-subject randomized crossover laboratory protocols with consecutive days of either simulated day work (circadian alignment) or simulated night work (circadian misalignment) allowed us to unveil the acute effects of circadian misalignment on perceived mood and well-being. Given such  acute effects in both non-shift workers and chronic shift workers, one may speculate that chronic exposure to circadian misalignment may be one additional key factor leading to the onset of depression in shift workers.

Lastly, while we observed that circadian misalignment decreased sleep efficiency in both non-shift workers and shift workers, no significant associations occurred between sleep efficiency (and other sleep parameters) and mood in either groups, possibly due to the sample size. Chronic sleep disruption is associated with negative emotions^53^, and chronic poor sleep quality has also been shown to delay affective recovery from negative events^54^. It is now well-established that shift workers often experience shorter sleep duration, increased frequency of napping, and greater variability of their sleep times^27,55^. This can lead to disrupted sleep/wake stability, which in turn can cause long-term circadian misalignment with ramifications to mental health and disease.

Potential limitations to our studies include the limited sample size (despite the within-subject design in each experiment), which warrants future studies with larger sample sizes to determine the magnitude of circadian misalignment effects on emotional state in shift workers. Furthermore, our analyses did not account for potential group effect of circadian phase or individual differences in circadian phase as indexed by, e.g., DLMO. Analyses of DLMO were not performed due to the limited sample size in each experiment and because light levels were ~ 90 lx during circadian alignment and misalignment. Exposure to 90 lx can result in ~ 50% of the maximum phase shifting capacity and melatonin suppression^56^, and recent evidence shows that melatonin suppression can occur at much lower light levels^39,57^. Thus, the effects of circadian phase as well as individual differences in the adverse mood consequences of circadian misalignment remain to be established. For the former, the circadian misalignment effects on mood and well-being might reflect the effects of a system that may be slowly or minimally re-entraining. Moreover, it might be likely that participants had negative mood levels during the biological night irrespective of a circadian misalignment effect per se. Future studies would be required to test these possible biological underpinnings as the current study design precludes testing such effects.

Despite decades of research on the impact of shift work on mental health, it remains unclear how shift work—in its chronobiologically most marked form, i.e., night work—can increase the risk of developing or exacerbating mood disorders. Collectively, our findings help to establish circadian misalignment as an important—and previously unrecognized—biological contributor for mood vulnerability in shift work settings. By identifying the role of circadian misalignment on emotional state, we can ultimately develop simple, yet effective evidence-based sleep/circadian interventions (e.g. appropriate light exposure, sleep hygiene) to improve the quality of life—an essential requirement for optimal mood— of shift workers.
